# Artificial intelligence–driven multimodal fusion for precision diagnosis and personalized management of breast cancer

**DOI:** 10.3389/or.2026.1849152

**Published:** 2026-06-15

**Authors:** Mingyu Zhang, Juhang Chu, Zixin Wang, Yaru Wang, Luyao Huang, Mingping Qian

**Affiliations:** Department of General Surgery, Shanghai Tenth People’s Hospital, School of Medicine, Tongji University, Shanghai, China

**Keywords:** artificial intelligence, breast cancer, digital twin, minimal residual disease, multimodal fusion

## Abstract

Breast cancer is the most common malignancy among women worldwide, characterized by pronounced heterogeneity across molecular profiles, imaging phenotypes, and the tumor microenvironment. As precision oncology continues to advance, diagnostic strategies that rely predominantly on single-modality imaging or pathology face inherent limitations in early detection, individualized risk stratification, and timely recurrence assessment. Recent progress in artificial intelligence, particularly deep learning–based methods, has accelerated the development of multimodal fusion models that integrate radiomics, digital pathology, multiomics data, liquid biopsy biomarkers such as circulating tumor DNA, and clinical variables within unified computational frameworks. These integrative approaches enable the discovery of cross-modal associations and have demonstrated improved performance in diagnosis, molecular subtyping, treatment response prediction, and prognostic evaluation. In this review, we provide a comprehensive overview of the fundamental principles, methodological advances, and representative clinical applications of AI-driven multimodal fusion in breast cancer. We further discuss emerging directions, including digital twin–based modeling, dynamic monitoring of minimal residual disease, and multimodal large language models. Finally, we highlight current challenges related to data standardization, model interpretability, and multi-center validation, and outline future perspectives toward clinically translatable and robust intelligent systems.

## 1 Introduction

Breast cancer remains the most common malignancy among women worldwide, with a continuously rising incidence and a noticeable trend toward younger onset (1). Its marked molecular heterogeneity further complicates clinical management. In current practice, precision diagnosis and treatment of breast cancer rely on a combination of imaging modalities—including mammography (MG), ultrasound (US), magnetic resonance imaging (MRI), and digital breast tomosynthesis (DBT)—together with pathological assessment based on conventional hematoxylin–eosin and immunohistochemical staining, whole-slide imaging, and molecular biomarker profiling such as estrogen receptor (ER), progesterone receptor (PR), human epidermal growth factor receptor 2 (HER2), and Ki-67 proliferation index (Ki-67).

Nevertheless, several critical challenges persist throughout real-world clinical workflows. Early detection, particularly in women with dense breast tissue, remains limited by a substantial false-negative rate. The assessment of axillary lymph node (ALN) metastasis still depends largely on intraoperative or postoperative pathology, leaving preoperative decision-making insufficiently informed. Distinct molecular subtypes exhibit heterogeneous responses to neoadjuvant chemotherapy (NAC), yet current tools struggle to accurately predict pathological complete response before treatment. Postoperative surveillance also faces delays in identifying recurrence, especially in cases of occult relapse or minimal residual disease (MRD). Moreover, imaging, pathology, and multiomics information often exist as highly fragmented, cross-scale datasets, with substantial heterogeneity between platforms and institutions, and lacking a unified integrative framework. Under these circumstances, diagnostic and therapeutic strategies based predominantly on single-modality evidence are increasingly inadequate to support precise, individualized decision-making across the continuum of breast cancer care—from early screening and risk stratification to treatment selection and long-term follow-up. Establishing a framework capable of integrating heterogeneous multimodal data and applicable to real-world clinical scenarios has therefore become essential for advancing contemporary management paradigms.

Artificial intelligence (AI), designed to emulate complex tasks traditionally performed by human experts, has rapidly developed in the domains of medical data mining and high-dimensional feature learning, offering new methodological solutions to longstanding clinical challenges. AI methods can be broadly categorized into supervised, unsupervised, and reinforcement learning, each providing distinct computational strategies for pattern discovery and predictive modeling. Machine learning (ML) and deep learning (DL) serve as the algorithmic foundations that operationalize these paradigms across imaging, pathology, and multiomics data. ML approaches can synthesize diverse clinical information for risk prediction, classification, and decision support. DL models, built upon multilayer neural network architectures, excel in capturing latent representations from high-dimensional imaging data, decoding multiomics signatures, and linking histopathological phenotypes with molecular alterations (2).

**FIGURE 1:**
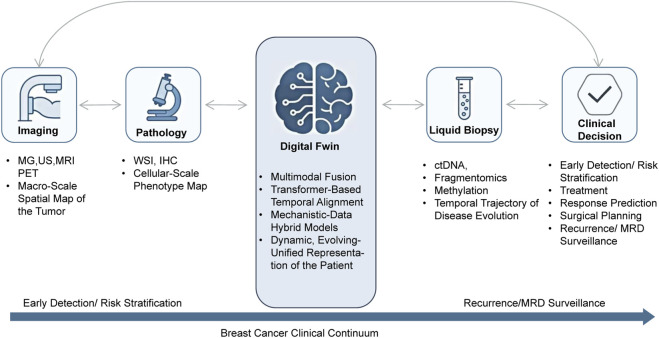
AI-driven multimodal fusion in breast cancer precision care. Schematic overview of multimodal data integration across the breast cancer clinical continuum. Imaging, digital pathology, multiomics, and liquid biopsy data are integrated through multimodal AI frameworks and potential digital-twin architectures to support risk stratification, treatment planning, and disease monitoring. MG, mammography; US, ultrasound; MRI, magnetic resonance imaging; PET, positron emission tomography; DBT, digital breast tomosynthesis; WSI, whole-slide imaging; IHC, immunohistochemistry; ctDNA, circulating tumor DNA; MRD, minimal residual disease.

In recent years, multimodal fusion techniques have witnessed remarkable progress, enabling the combined modeling of imaging, digital pathology, multiomics data, and clinical variables within a unified computational framework. Such approaches can uncover cross-modal and cross-scale associations that are inaccessible to traditional analytic methods. These advances are progressively reshaping key components of breast cancer management, including early detection, molecular subtyping, prediction of therapeutic response, treatment monitoring, and long-term prognostication (3,4). Building upon these developments, this review synthesizes current advances in AI-driven multimodal fusion for breast cancer precision medicine and highlights emerging but still exploratory directions, including digital twin modeling and dynamic monitoring of MRD (Figure 1). Our aim is to outline a conceptual and technical roadmap for transitioning breast cancer management from experience-based to data-driven precision care. We first summarize key data modalities and fusion paradigms, then review representative clinical applications across the management continuum, and finally discuss translational challenges and future directions.

## 2 Technical principles of AI-Driven multimodal fusion

### 2.1 Common data modalities in breast cancer

Breast cancer research and clinical decision-making rely on a broad spectrum of multimodal data, among which medical imaging represents the most fundamental and indispensable component. MG remains the cornerstone of global breast cancer screening due to its high level of technological maturity and wide accessibility, and it is particularly irreplaceable for the detection of microcalcifications. US, characterized by its real-time, non-invasive, and convenient nature, is especially suitable for Asian women and individuals with dense breast tissue; it is commonly applied for preliminary lesion evaluation and assessment of axillary lymph-node status. MRI, with its superior soft-tissue contrast, enables detailed visualization of tumor morphology, hemodynamic characteristics, and spatial relationships with surrounding structures. As such, MRI serves as a critical tool for local staging, monitoring response to NAC, and screening high-risk populations. DBT, by mitigating tissue-overlap artifacts, significantly improves the detection rate of small lesions and has gradually emerged as an important complement to conventional MG. Positron emission tomography–computed tomography (PET-CT) and PET-MRI provide metabolic information of tumors, offering unique advantages in identifying distant metastases, monitoring disease recurrence, and characterizing complex or ambiguous lesions (5).

Laboratory diagnostic data are likewise crucial in the clinical management of breast cancer. Serum tumor markers such as CA15-3 and CEA may be elevated in a subset of patients and can offer indicative value for disease surveillance and recurrence risk assessment; however, their diagnostic specificity is limited, and major clinical guidelines do not recommend their use as standalone tools for follow-up decision-making (6).

In parallel, biomarkers including hormone receptor status, HER2 expression, and Ki-67 provide direct and essential information for molecular subtyping and therapeutic stratification. With the advancement of digital pathology, deep computational analysis of whole-slide image (WSI) enables quantitative characterization of nuclear heterogeneity, invasive margins, and the spatial distribution of tumor-infiltrating lymphocytes (TILs), and further supports tasks such as assessing HER2/ER status and predicting lymphovascular invasion (LVI) or ALN involvement. A growing body of systematic reviews and methodological studies has demonstrated the potential of AI-enhanced WSI analysis to improve diagnostic consistency, prognostic evaluation, and workflow efficiency, while also underscoring the need for robust external validation and standardized evaluation frameworks (7).

The rapid evolution of multiomics technologies has enabled in-depth characterization of breast cancer across multiple molecular layers, including mRNA transcriptomics, key mutational landscapes (such as BRCA1/2, PIK3CA, and TP53), proteomics, and metabolomics. The scale and analytical resolution of these data have reached unprecedented levels, making them indispensable tools for delineating tumor molecular heterogeneity, identifying prognostically relevant signatures, and guiding individualized therapeutic strategies.

Notably, the advantages of multimodal synergy have already been demonstrated in real-world radiogenomic investigations. A recent MRI-based radiogenomic analysis integrating DCE-MRI radiomics with transcriptome-derived gene-expression signatures in 111 TCGA/TCIA breast cancers—supplemented by an external validation cohort of 15 cases—showed that the fused model achieved area under the receiver operating characteristic curve (AUC) values of 0.84, 0.75, and 0.82 across training, internal testing, and external datasets, respectively, exceeding the performance of radiomics-only and genomics-only models (8). Complementary evidence from another deep radiogenomic framework, which extracted 4,096 autoencoder-derived radiomic features from the MRI scans of 110 patients and linked them to multilevel genomic programs, demonstrated that more than 1,000 imaging features were significantly correlated with risk genes, pathway activities, and molecular signatures. These deep radiomic phenotypes further achieved AUCs >0.90 in predicting ER, PR, HER2 status and T/N categories, markedly outperforming handcrafted radiomics (9). Emerging subfields such as ultrasound-based radiogenomics have also provided quantitative evidence supporting genotype prediction directly from US-derived tumor phenotypes. In one dataset consisting of 386 breast cancers, incorporating two-dimensional and Doppler ultrasound features with molecular subtype increased the AUC for PIK3CA mutation prediction from 0.553 to 0.610 (single predictors) to 0.741 in the training cohort and 0.715 in independent validation. The same analysis also identified strong associations between specific subtypes—such as Luminal B/HER2+, HER2+/ER–, and TNBC—and TP53 mutation status (10). A complementary deep-learning approach using an enhanced ResNet architecture trained on 800 ultrasound images from 312 patients achieved an AUC of 0.775 for PIK3CA mutation classification, outperforming conventional machine-learning classifiers and several standard CNN architectures (11). In addition, small prospective studies and multiomics ctDNA analyses have suggested that plasma circulating tumor DNA signals may detect molecular relapse months to years before clinical recurrence, correlating with treatment response and enabling more personalized surveillance trajectories (12).

Collectively, these findings underscore that AI-enabled integration of imaging, pathology, multiomics, and clinical information has the potential to transcend the limitations of any single modality and establish new avenues for precision diagnosis and treatment in breast cancer. However, many of these studies remain retrospective or exploratory, with modest cohort sizes, heterogeneous data acquisition protocols, and limited external validation, warranting cautious interpretation before broader clinical translation.

### 2.2 Multimodal fusion strategies

**FIGURE 2:**
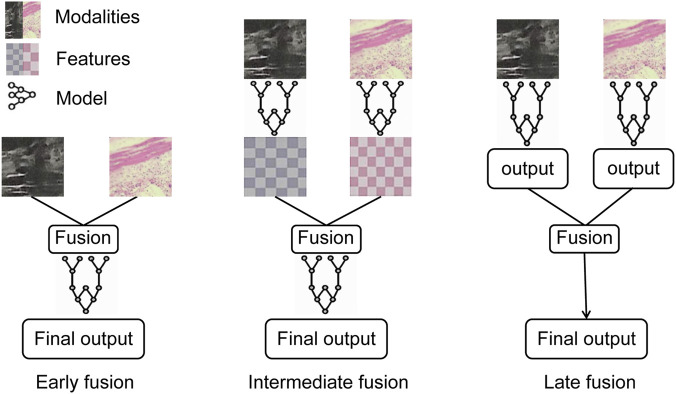
Multimodal fusion paradigms in AI-driven breast cancer analysis. Overview of multimodal fusion strategies based on the stage of data integration. Early fusion combines raw inputs, intermediate fusion integrates feature representations, and late fusion aggregates predictions from independent models.

Multimodal data fusion in breast cancer aims to jointly extract complementary information from imaging, pathology, multiomics, and clinical records, thereby enhancing diagnostic accuracy, treatment-response prediction, and recurrence-risk assessment through cross-modal learning. According to the stage at which fusion occurs, existing methods can be categorized into early fusion, late fusion, and intermediate fusion (Figure 2).

#### 2.2.1 Early fusion

Early fusion concatenates raw data from different modalities at the input layer before feeding them into a unified model. This approach preserves the full information content of modalities such as MG, US, MRI, DBT, WSI, and various omics datasets while simplifying the overall model architecture to some extent. Theoretically, it avoids the loss of fine-grained details that may occur during early feature extraction and facilitates cross-modal co-modeling at the pixel or signal level.

However, substantial discrepancies among breast cancer modalities—including scale differences between imaging and transcriptomics, the multi-sequence nature of MRI, and the extremely high resolution of WSIs—lead to considerable challenges in data preprocessing. Early fusion is also highly sensitive to noise and modality missingness, and often lacks deep feature-level interactions across modalities. Furthermore, the large number of trainable parameters and the need to retrain the entire model when introducing new modalities limit its practicality in clinical research. Schouten et al. (13) found that, among 432 multimodal AI studies in medicine, early fusion was used in only 6% of cases—substantially lower than the adoption of intermediate fusion (79%) and late fusion (14%).

#### 2.2.2 Late fusion

Late fusion independently models each modality and integrates their predictions at the decision layer through majority voting, weighted averaging, Bayesian fusion, or other ensemble strategies. This design allows MG, MRI, US, WSI, ctDNA, and electronic health record (EHR) data to use modality-specific architectures without requiring input alignment, offering clear advantages in multi-center settings, data collected at different time points, or scenarios with substantial distributional heterogeneity. Late fusion also demonstrates strong robustness by mitigating the influence of low-quality or abnormal modalities on the overall results. Nevertheless, because interactions occur only at the prediction stage, cross-modal complementarities are not fully exploited, and the degree of information integration remains limited. As a result, late fusion often underperforms in tasks requiring fine characterization of the tumor microenvironment, treatment-response dynamics, or complex histopathological patterns.

#### 2.2.3 Intermediate fusion

Intermediate fusion has become the most widely adopted strategy in oncology AI research, particularly for modeling the complex cross-modal heterogeneity of breast cancer. Its core principle is to first extract modality-specific features—using architectures such as ResNet for WSI, Swin Transformer for MRI, 1D-CNN for ctDNA, and MLPs for clinical variables—and subsequently perform deep cross-modal interaction at the feature level. Techniques such as attention mechanisms, cross-modal alignment, and graph-based modeling are commonly employed to capture latent relationships across modalities.

Intermediate fusion preserves modality-specific representations while simultaneously learning high-level semantic associations in a shared embedding space, enabling superior performance across multiple breast cancer tasks. Nakach et al. (14) systematically reviewed 47 multimodal deep-learning studies for breast cancer classification published between 2018 and 2023, analyzing modality combinations, fusion strategies, model architectures, and performance metrics. Their results demonstrated that intermediate fusion is more prevalent and consistently achieves higher AUCs than early or late fusion. As such, intermediate fusion has emerged as the dominant technical paradigm for intelligent breast cancer diagnosis and treatment.

### 2.3 Learning paradigms in AI-based breast cancer management

In real-world multimodal breast cancer applications, supervised learning forms the backbone of most clinically deployed AI models. ML methods such as logistic regression, random forests, and support vector machines feature simple architectures, strong interpretability, and robustness with small-to-medium datasets, making them valuable for risk scoring, molecular subtype prediction, and clinical decision support (15). DL models—including convolutional neural networks (CNNs), recurrent neural networks (RNNs/LSTMs), and Transformers—enable end-to-end extraction of high-dimensional multimodal features and have demonstrated substantial success in automated image segmentation, histopathological classification, and recurrence-risk prediction. However, despite their high accuracy, the inherent “black-box” nature of deep models still limits clinical adoption (16). Explainable AI (XAI) methods such as LIME, SHAP, and Grad-CAM provide visualized explanations and feature-attribution analyses across imaging, omics, and structured clinical data, thereby enhancing model transparency and clinical trustworthiness. However, current interpretability approaches still face several practical limitations. Many XAI methods provide *post hoc* explanations that may not reliably reflect the reasoning process learned by deep-learning models. Saliency-based visualizations can also be sensitive to model architecture, data distribution, preprocessing pipelines, and input perturbations, limiting reproducibility and clinical consistency (17). In addition, feature-attribution methods primarily identify statistical associations rather than causal mechanisms, making it difficult to determine whether highlighted regions or features truly drive clinically meaningful predictions. In multimodal settings, interpretability becomes even more challenging because contributions from imaging, pathology, genomics, and clinical variables may interact in highly nonlinear ways that are difficult to disentangle. Moreover, explanations considered visually plausible do not necessarily correspond to clinically valid reasoning, and different XAI methods may generate inconsistent interpretations for the same prediction. Therefore, current XAI approaches should be interpreted cautiously and evaluated through standardized, clinically grounded frameworks, rather than being treated as direct evidence of model trustworthiness (18).

Unsupervised learning approaches—including PCA, k-means clustering, t-SNE, and generative adversarial networks (GANs)—uncover latent structures within high-dimensional unlabeled data through clustering, dimensionality reduction, or anomaly detection. These methods have proven useful in identifying molecular subtypes, characterizing tumor-microenvironment heterogeneity, and discovering imaging–omics associations (19).

Reinforcement learning (RL), based on a “state–action–reward” mechanism, has been explored for adaptive treatment optimization, although most current applications in oncology remain simulation-based rather than clinically validated (20).

**FIGURE 3:**
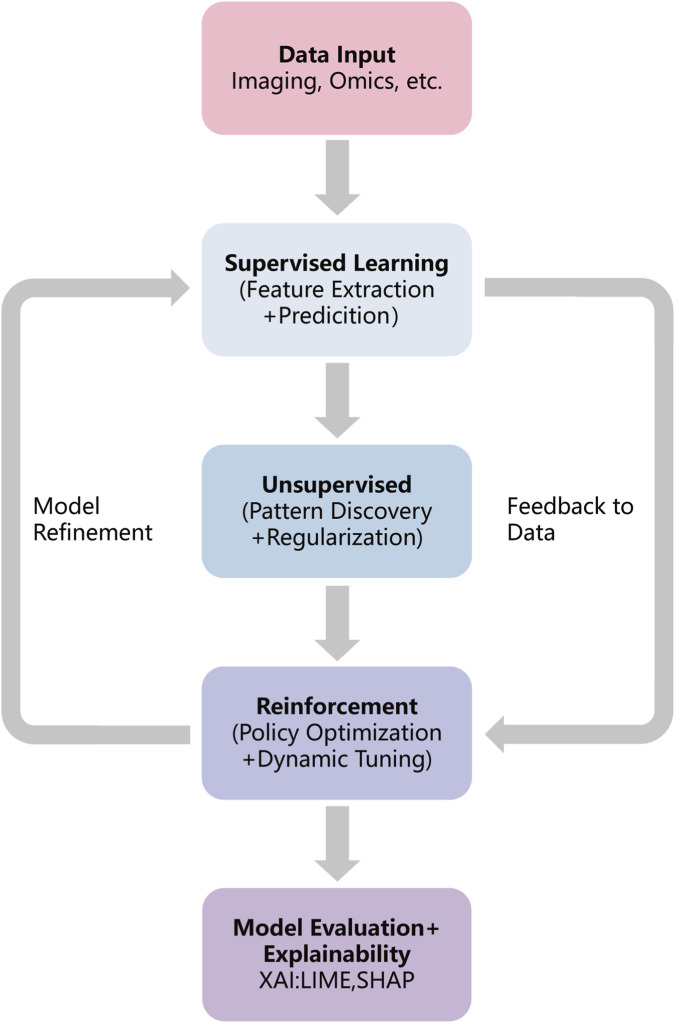
Hybrid learning frameworks in AI-based breast cancer diagnosis and management. Representative hybrid artificial intelligence architectures integrating supervised, unsupervised, and reinforcement learning to enhance multimodal analysis and model performance. Explainable artificial intelligence techniques are incorporated to improve interpretability. AI, artificial intelligence; XAI, explainable artificial intelligence; LIME, local interpretable model-agnostic explanations; SHAP, SHapley Additive exPlanations.

To improve robustness and interpretability, hybrid learning systems combining supervised prediction, unsupervised pattern discovery, and limited sequential decision modeling have been investigated as potential frameworks for multimodal integration in breast cancer (Figure 3).

At the imaging level, Qasrawi et al. (21) proposed a hybrid ensemble model based on MG data using a “preprocessing enhancement + multi-network ensemble” strategy. Tested on approximately 20,000 mammograms with clinical validation on 800 cases, the model achieved an overall accuracy of about 99.7%, although further external validation remains necessary. Similarly, Lilhore et al. (22) introduced a CNN-biLSTM hybrid network that achieved around 99.2% accuracy on the CBIS-DDSM and MIAS datasets and used saliency-map visualization to improve clinical interpretability. However, such high reported accuracies should be interpreted with caution, as dataset overlap, limited external testing, class imbalance, and model overfitting may inflate apparent performance in retrospective benchmark settings. Looking ahead, hybrid and cascaded architectures are expected to evolve toward unified multimodal frameworks that leverage federated learning, self-supervision, and causal inference to improve generalizability and trustworthiness across diverse clinical environments.

## 3 AI-based multimodal integration across the breast cancer management continuum

### 3.1 Early screening and diagnosis

#### 3.1.1 Multimodal imaging diagnosis

Early detection is vital for reducing breast cancer mortality, but MG remains limited in dense breasts. Consequently, recent studies have evaluated whether AI-assisted MG or multimodal imaging can enhance screening performance. Large real-world evaluations now show that AI-assisted screening improves detection performance and reduces variability across radiologists.

In the Korean National Breast Cancer Screening Program, Chang et al. (23) analyzed data from 24,543 women and compared standalone radiologist reading with AI-based computer-aided detection (AI-CAD)–assisted interpretation. The AI-assisted workflow improved the cancer detection rate from 5.01‰ to 5.70‰ without significantly altering the recall rate (p = 0.564), suggesting that AI can enhance sensitivity without increasing unnecessary recalls. Luo et al. (24) developed a Mixture-of-Modality-Experts (MOME) framework using multiparametric MRI data from 5,205 cases, which performed feature-level fusion of multiple MRI sequences. MOME significantly outperformed single-sequence models in lesion detection and subtype prediction, with attention mechanisms enhancing inter-modality complementarity and compensating for missing modalities. Similarly, van Winkel et al. (25) conducted a simulation study using 42,236 mammographic images from the Dutch screening program, showing that an AI system acting as an independent “second reader” increased sensitivity by about 8.4% while maintaining a comparable cancer detection rate—indicating that AI could reduce human workload and improve screening efficiency. Oviedo et al. (26) further developed an interpretable AI model for early MRI screening that employed Grad-CAM heatmaps for lesion localization, achieving an AUC of 0.84 (external validation 0.86), superior to conventional deep networks and providing improved transparency and traceability in Breast Imaging Reporting and Data System (BI-RADS) classification. Overall, multimodal AI—from intra-modality “multi-sequence fusion” to workflow-level “AI–human collaboration”—has demonstrated combined advantages in sensitivity, stability, and clinical trustworthiness for early screening. Nevertheless, its generalizability across scanners and populations still requires validation in large prospective studies.

#### 3.1.2 Integration of imaging with clinical and genomic data for precision diagnosis

In real-world clinical settings, imaging findings alone are often insufficient to achieve precise risk stratification, particularly given the heterogeneity of breast cancer phenotypes. Key patient-specific variables—such as age, breast density, hereditary susceptibility (e.g., BRCA1/2 mutation status), and endocrine milieu—critically shape disease presentation and must therefore be integrated with imaging information. Feature-level multimodal fusion frameworks enable the joint modeling of structured clinical variables and high-dimensional imaging representations, thereby improving the robustness of molecular subtype classification and individualized risk prediction.

Evidence from radiomics-based investigations supports the value of such multimodal integration. In a cohort of 190 patients with invasive ductal carcinoma, a fusion model incorporating three-dimensional DCE-MRI radiomic signatures with clinical attributes demonstrated clinically meaningful discriminative ability across major molecular subtype tasks. The model achieved an AUC of 0.903 for distinguishing TNBC (triple-negative breast cancer) from non-TNBC, while classification performance for Luminal and HER2-enriched disease also exceeded AUC values in the range of 0.812–0.837. These findings underscore the promise of MRI–clinical information fusion as a non-invasive strategy for subtype prediction (27). Beyond radiomics, the MOME framework further illustrates the extensibility of deep multimodal fusion. Through the use of modality-specific experts and adaptive weighting mechanisms, MOME captures complementary representations across multiple MRI sequences and enables dynamic interaction between modalities. Although primarily developed for imaging-based diagnostic tasks, its architectural design is inherently compatible with the incorporation of clinical indicators, pathological characteristics, and even transcriptomic markers, offering a methodological foundation for future imaging–genomic integrative modeling (24).

Taken together, intermediate-level fusion of imaging, clinical, and molecular features has the potential to overcome the performance ceiling of single-modality models, providing a unified analytical pathway for early-stage subtype identification and personalized risk estimation. Nonetheless, several barriers must be addressed before clinical translation can be realized, including multi-center data harmonization, modality-missingness handling, cross-vendor variability, and privacy-preserving data governance.

#### 3.1.3 Integration of liquid biopsy data for early cancer detection

Liquid biopsy provides a molecular pathway for non-invasive early screening of breast cancer that is independent of imaging-based modalities. The fragmentomic features of ctDNA and circulating free DNA (cfDNA)—reflecting genome fragmentation patterns, nucleosome positioning, and tissue-of-origin signals—offer highly sensitive indicators of nascent tumor activity.

Recent multicenter evidence has demonstrated that cfDNA fragmentomics, combined with machine-learning algorithms and low-depth whole-genome sequencing, can effectively discriminate malignant from benign breast nodules. In a cohort of 613 female participants, a fragmentomics-based assay achieved a specificity of 94.1% when sensitivity was fixed at 90%. Diagnostic performance further improved when fragmentomic features were integrated with radiological assessments (AUC = 0.93–0.94) or with cfDNA methylation signatures (AUC = 0.96), underscoring the promise of fragmentomics-enabled AI models as scalable tools for early detection (28). At the methodological level, Helzer et al. (29) further demonstrated that several key fragmentomic metrics can be reliably reproduced using exon-based targeted sequencing panels, thereby establishing the feasibility of panel-level fragmentomics and laying a foundation for clinical translation.

Together, liquid biopsy and imaging may form a complementary “dual-channel” screening strategy: liquid biopsy provides systemic molecular signals, while imaging contributes spatial localization and phenotypic characterization. AI-driven fusion of these heterogeneous data streams may enhance the identification of occult lesions and MRD, particularly in dense breast tissue or biologically aggressive subtypes. Despite these advances, fragmentomic analysis remains in an exploratory phase. Standardization across laboratories, management of cross-platform batch effects, reduced assay sensitivity at very low ctDNA fractions, false-positive risks associated with clonal hematopoiesis (CHIP), and uncertainties surrounding cost-effectiveness continue to present major translational challenges. Robust validation in real-world screening populations will be essential before routine clinical adoption can be achieved.

### 3.2 Surgical planning and prediction of axillary lymph node risk

The status of the ALN is a critical determinant in formulating surgical strategies for breast cancer. It guides the need for ALN dissection (ALND) or sentinel lymph node biopsy (SLNB), influences decisions regarding adjuvant radiotherapy and chemotherapy, affects the choice of surgical approach, and plays a substantial role in long-term prognostic evaluation. Conventional ALN assessment relies on US, MRI, and intraoperative frozen-section pathology; however, these approaches suffer from limited sensitivity, operator dependence, and suboptimal preoperative risk stratification. In recent years, multimodal AI frameworks integrating imaging, digital pathology, clinical variables, and genomic features have markedly improved the stability and accuracy of ALN metastasis prediction, providing new methodological avenues for surgical planning and precision axillary management.

#### 3.2.1 Multimodal imaging–based prediction of ALN metastasis

Feature-level fusion of imaging modalities enables structural information derived from US, MRI, and DBT to complement one another, thereby overcoming the limitations inherent to any single modality. A retrospective study including 401 breast cancer patients fused US and MRI features with clinicopathologic variables such as ER/HER2 status and tumor size, and applied a naïve Bayes classifier with SHAP-based interpretability (30). The model achieved an AUC of 0.902 in the independent validation cohort—significantly outperforming all single-modality approaches. In clinical scenarios where histological information is difficult to obtain preoperatively, imaging–clinical fusion can provide reliable stratification before SLNB and help prevent unnecessary axillary interventions.

#### 3.2.2 Imaging–pathology fusion for ALN prediction

WSI capture microstructural characteristics including tumor architecture, invasive margins, nuclear heterogeneity, and the spatial distribution of TILs, which naturally complement imaging features. A recently published study employed the METACANS model to integrate WSI and clinical variables for preoperative ALN prediction (31). The study included 1,991 cases for training and 2,166 for external validation across five independent centers, demonstrating consistent generalizability. Furthermore, the model identified microstructural patterns strongly associated with metastatic risk—such as micropapillary formations and necrotic regions—highlighting that pathology–imaging fusion not only enhances predictive performance but also provides mechanistic interpretability.

#### 3.2.3 Prediction of positive surgical margins

Margin status during breast-conserving surgery directly determines the need for intraoperative re-excision and postoperative radiotherapy. Recent multimodal fusion models have been developed to predict margin positivity, with a clear emerging trend despite most studies remaining methodological. Evidence indicates that combining MRI—particularly DCE-MRI—with microstructural features extracted from WSI yields superior identification of patients at high risk for positive margins compared with unimodal imaging or pathology models (32). Current findings suggest that preoperative MRI + pathology/imaging fusion could reduce intraoperative uncertainty and refine surgical planning in breast-conserving procedures. Larger multicenter studies are anticipated to validate these early observations.

#### 3.2.4 Prediction of lymphovascular invasion

LVI is a key prognostic factor for local recurrence and distant metastasis, yet traditional assessment relies primarily on postoperative pathology, limiting its value in preoperative stratification. Multimodal AI models have shown promise in LVI prediction: WSI can quantify vascular structures, stromal reactions, and nuclear heterogeneity, whereas MRI contributes information on tumor margins, dynamic enhancement kinetics, and peritumoral stromal composition. One multiparametric MRI radiomics study integrating intratumoral and peritumoral features achieved an AUC of approximately 0.84 (33). Another retrospective study leveraged multiparametric MRI (including intra- and peritumoral regions) with radiomics and DL to build a model for LVI prediction, achieving an AUC of 0.872 in the validation cohort (34). Such imaging–pathology fusion frameworks may serve as valuable components of comprehensive preoperative risk assessment, aiding personalized axillary surgery and systemic therapy planning.

Multimodal AI research in axillary management has progressed from simple imaging fusion toward deeper integration of imaging, pathology, and clinical data. Among these, multicenter imaging–WSI fusion models currently represent the most mature evidence base. By contrast, multimodal prediction of margin positivity and LVI—reflecting subtle invasive phenotypes—remains predominantly in single-center methodological exploration, underscoring the need for broader institutional validation. In addition, Peng et al. (35) proposed a US + H&E-based fusion strategy achieving an AUC of approximately 0.70 in 211 patients. Although performance remains modest, this approach demonstrates feasibility when only limited preoperative tissue is available. The study further highlights that core-needle biopsy pathology can still serve as an effective modality alongside US/MRI to deepen risk assessment. Given challenges such as US standardization, heterogeneous pathology sources, and unresolved cross-modal alignment issues, this line of research—while not yet mature—presents a promising future direction for improving preoperative assessment, surgical planning, and individualized axillary management.

### 3.3 Prediction of treatment response

Treatment selection in breast cancer is fundamentally driven by the biological characteristics of the disease. Endocrine signaling, HER2 receptor activity, cell-cycle regulation, and the immune microenvironment collectively shape therapeutic sensitivity. Yet even within the same molecular subtype, substantial interpatient heterogeneity exists in response to endocrine therapy, HER2-targeted agents, CDK4/6 inhibitors, immunotherapy, and NAC. Traditional clinical markers or single-modality imaging approaches are insufficient to reliably distinguish responders from non-responders before treatment initiation. Emerging multimodal AI frameworks—integrating imaging, digital pathology, transcriptomics, gene-expression signatures, and liquid biopsy—offer a more comprehensive characterization of tumor functional states, thereby enabling more refined prediction of therapeutic response.

#### 3.3.1 Endocrine therapy

The response to endocrine therapy is primarily influenced by ERα conformational alterations, ESR1 mutations, and the activity of ER-associated transcriptional programs. Certain hotspot mutations (such as Y537S and D538G) enable ligand-independent activation of ERα, markedly diminishing the therapeutic efficacy of aromatase inhibitors. Large-scale transcriptomic analyses further show that tumors characterized by strong ER signaling and low proliferative activity tend to be more responsive to endocrine therapy, whereas those displaying a “low-ER, high-proliferation” pattern are more likely to exhibit intrinsic resistance (36).

From an imaging perspective, radiomic analysis based on multiparametric breast MRI—including DCE-MRI, T2WI, and DWI-ADC—has demonstrated strong ability to predict endocrine resistance in hormone receptor-positive (HR-positive) breast cancer, with radiomic–clinical models achieving AUCs above 0.90 in both internal and external validation cohorts (37). Together, ESR1 mutation profiles, ER-related transcriptional programs, and functional MRI dynamics constitute key multimodal components for predicting endocrine therapy response.

#### 3.3.2 HER2-targeted therapy

Spatial heterogeneity in HER2 expression has been established as a key biological determinant of response to anti-HER2 therapy. Quantitative proteomic and histologic analyses demonstrate that HER2 abundance exists along a continuous spectrum rather than a binary positive/negative state. Tumors with relatively lower HER2 levels, even within the HER2-positive category, are more likely to exhibit suboptimal therapeutic responses (38). These findings suggest that regional differences in HER2 expression may shape differential drug sensitivity and provide the mechanistic rationale for developing imaging-based or computational models to infer HER2 spatial patterns. With the advancement of high-throughput proteomic technologies, refined HER2 quantification is expected to become an important adjunct for patient stratification in future anti-HER2 treatment strategies.

#### 3.3.3 CDK4/6 inhibitors

Sensitivity to CDK4/6 inhibition is largely governed by the integrity of cell-cycle regulatory pathways, particularly the RB/E2F axis. Multiomics profiling reveals that high p16 expression and heterozygous RB1 loss are strongly associated with differential susceptibility to CDK4/6 blockade (39). Predictive models incorporating these cell-cycle program signatures outperform traditional markers such as Ki-67 or PAM50, highlighting the value of biologically grounded features for treatment stratification. Although further large-scale prospective validation is required, cell-cycle–based molecular signatures have become central to identifying patients who may benefit from CDK4/6 inhibitors.

#### 3.3.4 Immunotherapy

Prediction of immune checkpoint inhibitor response increasingly leverages the spatial organization of TILs, PD-L1 expression patterns, and dynamic ctDNA changes. The spatial organization of TILs reflects the immune architecture of the tumor microenvironment. Deep learning–based quantification of TIL spatial topologies on H&E slides correlates strongly with prognostic and immune-related molecular features, providing an interpretable foundation for predicting response to immune checkpoint inhibitors (ICIs) (40).

In contrast, evidence regarding ctDNA dynamics as a predictive marker for immunotherapy in breast cancer remains limited. However, robust data from other solid tumors indicate that early ctDNA clearance is strongly associated with higher objective response rates and prolonged progression-free and overall survival (41,42). These findings suggest that longitudinal ctDNA monitoring may provide an early signal of treatment trajectory in breast cancer and represents a promising area for future investigation.

#### 3.3.5 Neoadjuvant chemotherapy

In breast cancer, NAC is one of the most thoroughly investigated settings for multimodal response prediction. Multiparametric MRI—particularly quantitative DCE-MRI parameters, diffusion metrics, and texture-based features—has repeatedly been shown to correlate with pathological complete response (pCR). Radiomics models derived from multi-sequence MRI have demonstrated robust discrimination between pCR and non-pCR across multicenter cohorts, establishing MRI-based signatures as reliable imaging biomarkers of chemosensitivity (43).

Building on this foundation, cross-modal DL has begun to integrate biopsy H&E WSIs, longitudinal US examinations, and clinical variables. In a recent multicenter study, a cross-modal model achieved AUCs around 0.85 for pCR prediction in both validation and test cohorts; incorporating clinicopathologic features further increased performance to approximately 0.87–0.88 (44). Recent prospective work indicates that combining DCE-MRI parameters with apparent diffusion coefficient (ADC) values enables early prediction of pCR within the first few treatment cycles, with AUCs in the range of 0.85–0.89 (44). Looking ahead, integrating multi-timepoint and multimodal data within real-world clinical workflows may transform NAC response assessment from a one-off classification task into a genuinely dynamic, decision-support process.

**FIGURE 4:**
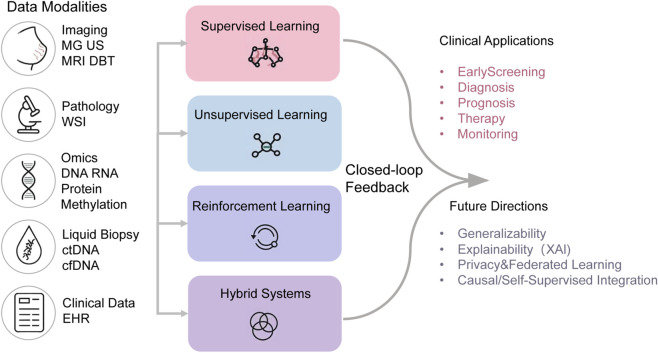
AI-driven multimodal integration for breast cancer management. Schematic representation of multimodal artificial intelligence applications across breast cancer management, including screening, diagnosis, treatment, and monitoring. Integrated learning frameworks enable clinical decision support and model refinement. MG, mammography; US, ultrasound; MRI, magnetic resonance imaging; DBT, digital breast tomosynthesis; WSI, whole-slide imaging; ctDNA, circulating tumor DNA; cfDNA, circulating free DNA; EHR, electronic health record; XAI, explainable artificial intelligence.

Multimodal AI enables the integration of structural imaging, cellular morphology, and molecular regulatory signals to achieve a more comprehensive representation of tumor biology, thereby improving prediction of therapeutic response across diverse treatment modalities (Figure 4). As interpretability improves, multi-center validation accumulates, and clinical workflows become increasingly compatible with data-driven tools, the translational potential of multimodal AI becomes increasingly evident. These technologies are poised to become an integral component of precision therapy decision-making in breast cancer. Nevertheless, current evidence remains dominated by retrospective datasets with heterogeneous imaging protocols, pathology workflows, and sequencing platforms. Large-scale prospective, multi-center trials will be essential to fully realize the clinical utility of multimodal treatment-response prediction.

## 4 AI-guided ctDNA dynamic profiling for enhanced MRD surveillance in breast cancer

### 4.1 ctDNA-based MRD as an early molecular warning system

Postoperative recurrence in breast cancer varies widely across individuals—some patients recur within 6 months, whereas others may relapse 5–10 years later. Conventional imaging surveillance relies on structural alterations and often lags behind true biological progression. In contrast, ctDNA-based MRD assessment can capture minute tumor-derived signals and has demonstrated substantial lead time over radiologic or clinical relapse.

Early seminal work by Garcia-Murillas and colleagues showed that personalized ctDNA tracking can anticipate relapse several months to a year before clinical detection, and ctDNA re-positivity strongly correlates with inferior relapse-free survival (45). Similarly, Coombes et al. (46) demonstrated that persistent ctDNA positivity is associated with an elevated risk of distant metastasis. The recent CloneSight study further extended these observations, revealing that ctDNA may emerge up to 68 months before relapse in high-risk HR + early breast cancer, with characteristic progressive kinetic trajectories (47). Together, these findings establish ctDNA dynamics as a powerful molecular early-warning system.

### 4.2 Temporal patterns of ctDNA reflect residual disease activity

Despite its promise, most MRD analyses still rely on dichotomous “detectable vs. undetectable” thresholds, overlooking the rich information embedded in ctDNA kinetic curves—such as fluctuation amplitude, velocity of increase, or clearance patterns—which likely reflect underlying clonal evolution and residual tumor activity. Advances in ML provide a compelling avenue to decode these high-dimensional temporal signatures. Moser’s review highlights how fragmentomic attributes—fragment length distributions, end-motif preferences, nucleosome positioning—can be robustly captured using ML/DL models to detect extremely low ctDNA abundance. Complementarily, Tsui and co-workers demonstrated that deep-learning frameworks can extract subtle cfDNA fragmentomic features that escape mutation-based assays, offering a new window into early clonal dynamics (48). These developments suggest that AI-driven temporal modeling—such as transformer-based or other sequence-learning architectures—may help characterize longitudinal ctDNA measurements as continuous “molecular risk trajectories”. However, most current ctDNA-AI studies remain exploratory and are often limited by small longitudinal cohorts, short follow-up durations, and insufficient prospective validation.

### 4.3 Integrating ctDNA dynamics with imaging and multidimensional biomarkers

Clinical observations further underscore the need for dual-modality temporal fusion between imaging and ctDNA. Some studies have shown that longitudinal ctDNA measurements tend to reflect overall tumor burden assessed by MRI during neoadjuvant treatment, and integrating ctDNA status with MRI-based functional tumor volume can yield more informative estimates of pathological response and subsequent recurrence risk than relying on imaging alone (49). In real-world surveillance, patients may exhibit radiologic stability despite persistent ctDNA positivity—suggesting micro-metastatic disease or emerging resistance—whereas others show rapid ctDNA decline preceding visible radiologic change. Although no published study has yet implemented an AI-based dual time-series model combining MRI (or US/DBT) with ctDNA dynamics in breast cancer, such an approach is technically feasible. A dual-channel transformer that aligns imaging and molecular trajectories through cross-attention mechanisms could learn characteristic asynchrony patterns and enhance early detection of subclinical progression.

Beyond single-analyte monitoring, emerging multiomics cfDNA analytics offer additional layers of biological information. Van TTV et al. (50) integrated mutation, fragmentomic, and methylation features to differentiate multiple cancer types and stages, while some research showed that a fragmentomics-based ML model can distinguish early breast cancer from healthy individuals (28), confirming the applicability of fragmentomics-AI approaches in breast cancer.

Together, these findings suggest that ctDNA monitoring is evolving from mutation-based detection toward a multidimensional paradigm incorporating temporal kinetics, fragmentomics, methylation features, and deep-learning models. However, AI-driven MRD systems remain exploratory, and their future clinical value will depend on reliable integration with imaging and longitudinal clinical data.

## 5 Digital twin–driven personalized management of breast cancer

The concept of a digital twin, originally developed in engineering and aerospace, refers to the construction of a synchronized “virtual clone” of a physical system within a computational environment, enabling real-time monitoring and predictive simulation. With the rapid advancement of multimodal medical data and computational oncology, digital twins have begun to enter precision oncology and are increasingly explored as a potential framework for individualized cancer care (51). In breast cancer—a disease characterized by pronounced spatial and temporal heterogeneity—the core aim of a digital twin is to integrate imaging, pathology, multiomics, liquid biopsy, and electronic health records into an evolving computational replica of each patient. This virtual model is designed to simulate tumor evolution, forecast treatment response, and support clinical decision-making in a dynamic and iterative manner.

### 5.1 Architecture and functional components of medical digital twins

**FIGURE 5:**
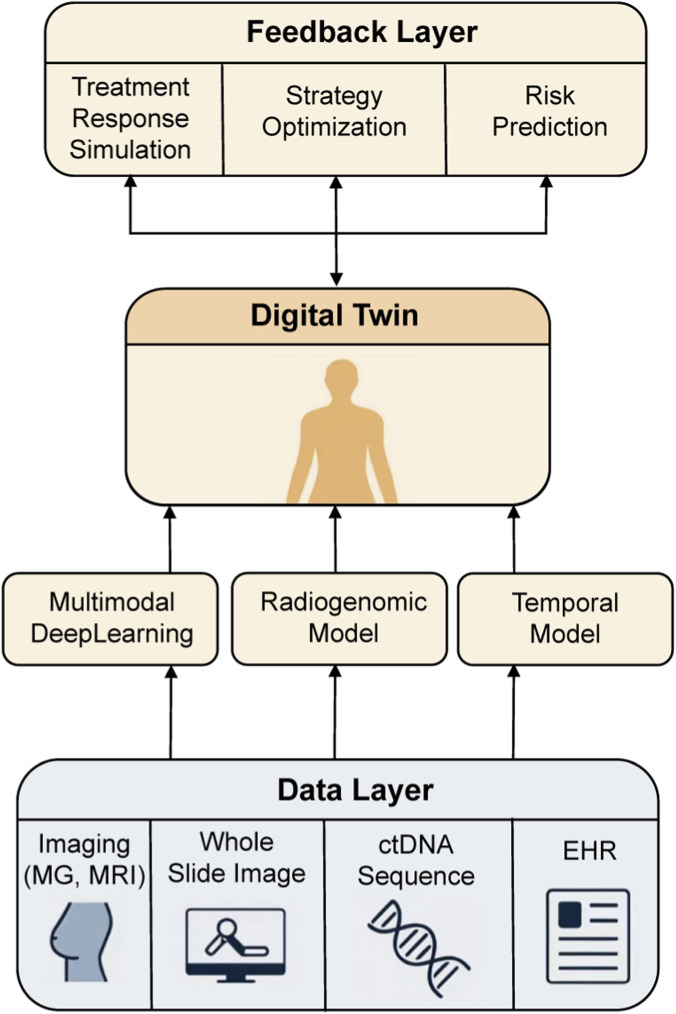
Framework of a digital-twin system for AI-driven breast cancer management. Conceptual framework of a digital twin integrating multimodal patient data with deep learning, radiogenomic, and temporal modeling approaches. The system enables dynamic simulation of disease evolution, treatment response prediction, and adaptive clinical decision-making through a feedback-driven architecture.

Current medical digital twin frameworks typically consist of three interconnected layers (Figure 5): the data layer, the model layer, and the feedback layer. The data layer serves as the foundation, incorporating high-dimensional multimodal inputs such as MG, US, MRI, and PET-CT; whole-slide pathology images; genomic, transcriptomic, and proteomic data; as well as ctDNA-based assays including fragmentomics and methylation profiling. Longitudinal electronic health records further refine the clinical context (52). Building on this foundation, the model layer employs multimodal DL, radiogenomics, mechanistic–data hybrid models, and temporal neural networks to represent tumor growth, clonal dynamics, and treatment-induced changes. A growing body of work has attempted to combine mechanistic tumor-growth equations and pharmacokinetic/biological priors with ML to improve interpretability and generalizability (53). The feedback layer translates model outputs into clinically relevant insights—such as predicted shrinkage curves under specific regimens, estimated risks of local or distant recurrence, toxicity–benefit trade-offs, and individualized long-term surveillance trajectories—thus forming a “data–model–decision” loop (54).

Across oncology, several comprehensive reviews have outlined the emerging landscape of digital twins. Moztarzadeh et al. (52) proposed a conceptual framework for cancer digital twins, highlighting the role of ML and multiscale modeling in reconstructing tumor response patterns and enabling virtual trials for chemotherapy, radiotherapy, and immunotherapy. Mollica et al. (51) emphasized that current evidence remains largely conceptual, with existing systems limited to small-scale prototypes rather than clinically validated platforms. More recently, Shen et al. (54) summarized practical implementations across tumor types, underscoring that the value of digital twins lies not only in constructing “virtual tumors,” but also in continuously recalibrating these models with multimodal real-world data to guide adaptive treatment decisions.

### 5.2 Early applications and emerging evidence in breast cancer

Within the field of breast cancer research, several early-stage explorations have been conducted. In one empirical study, researchers surveyed clinicians, healthcare administrators, and payers, finding broad optimism regarding the application of digital twin technology in personalized treatment planning, healthcare resource allocation, and clinical research. However, significant concerns were also raised, particularly regarding data privacy, regulatory standards, model transparency, and integration into existing clinical workflows (55). Although the study did not propose a computational system, it mapped out practical needs and barriers from multiple stakeholder perspectives. Zhao et al. (56) evaluated advances in imaging-based digital twin construction in Medical Physics, noting that high-resolution, multi-sequence breast MRI and DBT may serve as key imaging pillars for future breast digital twins. However, current work remains largely focused on organ-level or lesion-level modeling rather than full-pipeline digital replicas of breast cancer patients.

A particularly notable direction is the application of digital twins to neoadjuvant therapy in TNBC. Wu et al. (57) constructed MRI-driven digital twins based on longitudinal functional tumor-volume trajectories in patients receiving AC-T. These virtual models simulated alternative dosing schedules (weekly, biweekly, tri-weekly) to estimate individualized therapeutic benefit. Interestingly, the digital environment predicted substantial benefit from intensified regimens in some patients but not others, illustrating the potential of digital twins to support regimen selection. Although retrospective and limited to imaging data without integration of pathology or ctDNA, this study represents one of the first proofs of concept for digital-twin–guided neoadjuvant decision-making in breast cancer. Parallel developments in radiotherapy have explored tumor-dynamics–based digital twins to optimize dose distribution and fractionation strategies, laying technical foundations for future use in breast-conserving radiotherapy and partial breast irradiation.

### 5.3 Current limitations and developmental trajectories

Overall, no publicly available system currently integrates imaging, WSI, multiomics, ctDNA dynamics, and electronic health records into a single, clinically validated digital twin for breast cancer. Existing studies instead contribute individual components—tumor-dynamics modeling, multimodal fusion architectures, clinical workflow analysis, and prototype decision-support systems—that together outline the contours of a future breast cancer digital twin ecosystem. Thus, discussions of digital twins in breast cancer require both scientific rigor and cautious optimism: acknowledging the absence of mature clinical systems while clearly articulating a plausible developmental pathway. Technologically, the foundations established in multimodal AI, MRD (ctDNA) kinetic modeling, and transformer-based temporal architectures form the methodological substrate for next-generation digital twins (58). From a translational perspective, progress will depend on multi-institutional datasets, standardized data-sharing protocols, transparent model-validation frameworks, and regulatory pathways tailored to adaptive computational models. With these elements in place, digital twins may evolve from conceptual prototypes into clinically applicable decision-support systems for individualized breast cancer management. To provide a structured synthesis of the evidence discussed above, representative multimodal AI studies in breast cancer are summarized according to clinical task, integrated modalities, fusion strategy, validation design, evidence maturity, and current limitations (Table 1). This comparison highlights substantial heterogeneity in clinical maturity, ranging from exploratory radiogenomic, MRD, and digital-twin studies to more mature population-based AI screening investigations.

**TABLE 1:** Representative multimodal AI studies in breast cancer and their evidence maturity.

Domain	Clinical task	Study	Modalities integrated	Fusion strategy	Dataset/validation	Key findings	Evidence level	Current limitations
Radiogenomics	ALN metastasis prediction	Chen et al. (8)	MRI radiomics + transcriptomics	Intermediate fusion	111 TCGA/TCIA cases +15 external validation cases	Improved ALN prediction versus single-modality models	Moderate	Small external validation cohort
Radiogenomics	Molecular phenotype prediction	Liu and Hu (9)	MRI deep radiomic features + genomic profiles	Intermediate fusion	110 TCGA/TCIA patients	Associations between MRI features and molecular phenotypes	Exploratory–moderate	Retrospective association analysis
Ultrasound radiogenomics	TP53/PIK3CA mutation prediction	Huang et al. (10)	Ultrasound features + molecular subtype	Intermediate clinical–imaging fusion	243 training +143 validation patients	Improved mutation prediction using multimodal features	Moderate	Limited cross-platform generalizability
Screening and diagnostic stratification	AI-assisted mammography	Chang et al. (23)	Mammography + AI-CAD	Workflow-level AI assistance	24,543 women from a prospective multicenter screening cohort	Improved cancer detection without recall increase	High	Limited long-term implementation data
MRI diagnosis	Multiparametric MRI diagnosis	Luo et al. (24)	Multi-sequence breast MRI	Mixture-of-modality-experts/intermediate fusion	1,317 development +3,703 validation MRI examinations from three hospitals	Expert-level multiparametric MRI diagnosis	Moderate–high	Limited cross-population validation
Screening workflow	AI second-reader screening	Winkel et al. (25)	Mammography + AI second-reader simulation	Late/decision-level fusion	42,236 screening mammograms from a Dutch population-based screening cohort	Improved screening simulation performance	Moderate–high	Retrospective workflow simulation
MRI screening	Explainable MRI cancer detection	Oviedo et al. (26)	Breast MRI + explainable heatmaps	DL anomaly detection with XAI	9,738 MRI examinations +221 external validation examinations	Explainable MRI anomaly detection	Moderate	Limited clinical validation of explainability
Liquid biopsy	Early cancer detection	Liu et al. (28)	cfDNA fragmentomics + radiology	Fragmentomics-based machine learning	193 training +420 external validation participants from five cohorts	Improved breast nodule discrimination through multimodal integration	Moderate–high	Limited real-world screening validation
Axillary management	ALN metastasis prediction	Liu et al. (30)	US + MRI + clinicopathologic biomarkers	Multimodal clinical–imaging ML with SHAP	280 training +121 validation patients	Improved ALN prediction with SHAP interpretability	Moderate	Single-center retrospective design
Axillary management	Preoperative ALN prediction	Park et al. (31)	WSI + clinicopathologic data	Multimodal AI fusion	1,991 training +2,166 external validation cases from five cohorts	Externally validated ALN prediction using biopsy WSIs	Moderate–high	Moderate discrimination performance
Invasive phenotype	LVI prediction	Liu et al. (34)	Multiparametric MRI + radiomics + DL	Imaging feature fusion	183 training +79 validation patients	MRI-based multimodal LVI prediction	Moderate	Limited prospective validation
NAC response	pCR prediction	Liu et al. (43)	Multiparametric MRI + clinical information	Radiomics–clinical fusion	128 training +286 external validation patients from 3 cohorts	Superior pretreatment pCR prediction versus clinical models	Moderate–high	Limited prospective workflow validation
NAC response	Early pCR prediction	Guo et al. (59)	Biopsy WSI + multi-temporal US	Cross-modal deep learning	391 training +205 internal test patients from a single-center cohort	Early pCR prediction during NAC	Moderate	Single-center retrospective validation
MRD surveillance	Personalized ctDNA recurrence monitoring	Coombes et al. (46)	Patient-specific ctDNA assay	Personalized molecular tracking	49 patients with 208 serial plasma samples from a prospective multicenter cohort	Relapse detection up to 2 years before clinical recurrence	Moderate–high	Lack of standardized assay thresholds
Late relapse MRD	Ultrasensitive ctDNA monitoring	Comino-Méndez et al. (47)	Patient-informed ctDNA tracking	Longitudinal molecular monitoring	20 HR + BC patients with 55 serial plasma samples	Late relapse prediction up to 68 months before recurrence	Exploratory–moderate	Small exploratory cohort
MRD + imaging	NAC response and recurrence risk	Magbanua et al. (49)	ctDNA + MRI functional tumor volume	Dual-modality monitoring	84 patients from the multicenter I-SPY 2 trial	Correlated ctDNA/MRI tumor burden measures with added post-NAC prognostic value	Exploratory–moderate	Pilot study with limited sample size
Digital twin	NAC regimen optimization	Wu et al. (57)	Longitudinal MRI tumor-volume trajectories	MRI-based digital twin simulation	105 TNBC patients from the ARTEMIS trial	Simulated individualized NAC optimization with improved predicted pCR	Exploratory–moderate	Retrospective proof-of-concept simulation

ALN, axillary lymph node; ctDNA, circulating tumor DNA; DL, deep learning; MRD, minimal residual disease; NAC, neoadjuvant chemotherapy; SHAP, shapley additive explanations; TNBC, triple-negative breast cancer; WSI, whole-slide imaging.

## 6 Challenges and future directions

Although AI-driven multimodal fusion has shown considerable promise in breast cancer diagnosis and management—with meaningful advances in imaging analysis, digital pathology, ctDNA-based MRD surveillance, and early digital-twin prototypes—its translation into routine clinical practice remains constrained by several unresolved challenges. Foremost among these is the substantial heterogeneity across multimodal data sources, which continues to undermine model generalizability. Imaging protocols vary across vendors and institutions, with differences in scanner hardware, acquisition parameters, reconstruction algorithms, and image quality potentially introducing significant variability into radiomic and deep-learning features. In digital pathology, staining procedures, tissue preparation workflows, and scanner settings may generate substantial batch effects in WSIs, thereby affecting reproducibility across laboratories. Population-related bias also remains insufficiently addressed, as many current datasets are derived predominantly from specific geographic regions, ethnic populations, or highly selected tertiary-center cohorts, potentially limiting broader applicability. In addition, sequencing platforms differ in depth, chemistry, and preprocessing pipelines, further complicating cross-modal integration. Even when trained on large datasets, multimodal models frequently encounter distribution shifts in external cohorts, making cross-institutional deployment particularly difficult. The absence of internationally standardized multimodal breast cancer datasets—with harmonized imaging, pathology, molecular, and longitudinal clinical annotations—further limits robust benchmarking and external validation of AI systems.

A second barrier is the scarcity of large-scale, multi-center, systematically curated external validation cohorts. Most existing studies remain single-center or retrospective, and few datasets simultaneously integrate the full diagnostic spectrum—imaging, pathology, genomics, ctDNA dynamics, treatment data, and longitudinal outcomes. In contrast to fields with established population-level cohorts, breast cancer lacks internationally harmonized multimodal repositories, delaying the development of clinically reliable AI systems.

A third limitation involves the insufficient interpretability of deep multimodal architectures. While such models excel at capturing cross-modal associations and latent representations, their internal decision processes often remain opaque. Clinicians—especially in high-stakes scenarios such as surgical selection, neoadjuvant therapy adaptation, or early recurrence prediction—require explicit rationale to trust model outputs. Although XAI methods have improved visual highlighting and feature-attribution analysis, their integration into multimodal decision frameworks remains preliminary. Current interpretability techniques are often difficult to generalize across heterogeneous multimodal architectures, and there is still no consensus regarding how interpretability should be quantitatively evaluated or clinically validated in real-world oncology settings. Moreover, visually intuitive explanations do not necessarily guarantee biologically or clinically valid reasoning, further complicating physician trust and regulatory evaluation.

Furthermore, real-world clinical integration poses substantial systemic challenges. Deploying multimodal AI requires interoperable data standards, seamless interfaces with clinical information systems, well-defined regulatory pathways, and clear delineation of medico-legal responsibility. In addition, the high computational demands associated with multimodal foundation models, large-scale imaging analysis, and longitudinal data integration may limit implementation in resource-constrained healthcare settings. Economic considerations also remain insufficiently addressed, including reimbursement policies, cost-effectiveness evaluation, and long-term maintenance of AI infrastructure. From a clinical perspective, adoption may be hindered by limited model interpretability, increased workflow complexity, and variability in clinician trust and familiarity with AI-assisted decision systems. The use of highly sensitive data—ranging from high-resolution imaging to genomic and ctDNA profiles—further raises stringent requirements for privacy protection, cybersecurity, and ethical oversight. Achieving large-scale data sharing while maintaining confidentiality remains an unsolved institutional and policy challenge.

Looking ahead, the evolution of multimodal AI in breast cancer will depend not only on algorithmic innovation but also on building a sustainable translational ecosystem. A key priority is establishing an international breast cancer multimodal data-sharing consortium, enabling harmonized data governance and cross-center standardization. Parallel efforts should focus on developing unified preprocessing and normalization pipelines for imaging, pathology, and sequencing data to minimize inter-institutional variability. Multimodal large-language models (MLLMs) may support cross-modal information retrieval and clinical summarization in the future, although their role in breast cancer decision-making remains largely speculative and requires rigorous validation. Digital twin technologies may also facilitate more dynamic treatment-simulation frameworks, but their clinical applicability remains limited by the lack of prospective validation and standardized integration pathways. In parallel, combining AI with MRD monitoring through ctDNA kinetics, fragmentomics, and methylation signatures may further refine recurrence-risk assessment, although the clinical value of these approaches remains to be established in longitudinal real-world studies.

Overall, multimodal AI in breast cancer stands at a pivotal transition point. The next phase of progress will rely on coordinated advances in data infrastructure, regulatory frameworks, clinical validation, and interdisciplinary collaboration. AI will not replace clinicians; instead, it has the potential to become a foundational layer of future breast cancer management—supporting more precise, adaptive, and intelligent clinical decision-making.

## 7 Conclusion

This review provides a comprehensive overview of AI-driven multimodal fusion in breast cancer, highlighting advances in integrating imaging, digital pathology, multiomics profiles, liquid biopsy markers, and clinical data to support precise and individualized decision-making. Evidence to date demonstrates that multimodal models enhance performance across key clinical tasks—including early detection, molecular subtype characterization, prediction of treatment response, surgical planning, and MRD monitoring through ctDNA dynamics. The emergence of digital-twin frameworks further suggests a possible transition from static assessment toward more continuous, model-guided management throughout the disease course, although this remains an early translational direction.

Despite these advances, significant challenges remain, particularly in data heterogeneity, limited multicenter validation, model interpretability, workflow integration, and privacy governance. Future progress will depend on standardized multimodal data infrastructures, transparent and clinically aligned modeling frameworks, rigorous real-world evaluation, and sustained multidisciplinary collaboration. Overall, multimodal AI has the potential to become an important component of next-generation breast cancer care, enabling earlier detection, more accurate risk stratification, and more individualized therapeutic strategies.
